# Analyzing Chinese parents' and teachers' perception of play, children's emotional needs and therapy: implications for seeking help

**DOI:** 10.3389/fpsyg.2023.1219901

**Published:** 2023-09-08

**Authors:** Huiting Cao

**Affiliations:** Institute of Education, University College London, London, United Kingdom

**Keywords:** play therapy, Chinese parent's perception, mixed method, mental health, children's mental wellness, cultural conceptualization

## Abstract

**Introduction:**

Before going through play therapy with children, parents' consent is usually needed. Thus, Parents' perception of play therapy can be a very important variable that influences the extent to which children are exposed to play therapy. Previous research has demonstrated the significant influence of social-cultural factors on people's perceptions of play therapy. This may in turn influence parents' decision about whether their children will participate in play therapy. This study explores the factors that influence Chinese parents' decisions on their children's engagement in play therapy from a social-cultural perspective.

**Methods:**

This small-scale research includes the following: a questionnaire with 16 parents; three one-to-one semi-structured interviews with three parents; and one focus group with 3 teachers.

**Results:**

The results showed that Chinese parents' perceptions of therapy are mostly negative, which may reduce the desire of parents to let their children become involved with play therapy. The children's academic stress and the parents' economic pressures indicate a barrier for families to commit time and money to play therapy. Parents' confidence in their ability to recognize children's emotions, their lack of knowledge about play therapy, and the process of obtaining help from mental health services for children could be another barriers preventing engagement with play therapy.

**Discussions:**

Even though the research is conducted with a very limited sample size and the results cannot represent the wider population, this research provides some insights into this issue that can be explored in detail in future research, to re-evaluate the results and form a more concrete theory within a wider population in China.

## 1. Introduction

Play can be meaningful to children in many ways. Shrinivasa et al. (2018) suggested that play “is the method most children use to explore the world and to comprehend relationships, and it also helps them to understand the past and also to prepare for the future” (p. 82). In relation to this function of play for children, Howard (2010) further emphasized the importance of understanding the benefits of play, arguing children use play to connect to many aspects of their experiences and learning. The benefits of play are therefore inseparable from children's other activities.

According to Wood (2014), there has been a great deal of research showing evidence of the benefits of play in education. The benefits are not only related to the learning of academic subjects but also related to children's “social competence, wellbeing, positive orientations to learning and overall progression.” Other benefits of play in education have also been suggested. For example, the domain of play, particularly concerning object play, is considered to be the source of creativity. Jones (2020) asserts that play allows children to confront various challenges in a natural and spontaneous manner, which promotes growth and maturation.

Other benefits of play are usually related to children's emotional wellbeing. Play can be a way in which children express their emotions, and it could be very useful for children, especially young children who have not yet fully developed the verbal skills to express their emotions. Homeyer and Morrison (2008) explained the way children express their emotions through play: “[during] play, children can play out negative life experiences by breaking them into smaller parts, releasing feelings that accompany each part, assimilating each experience back into the view they have of themselves, and obtaining a new level of mastery.” Furthermore, Schaefer (2003) demonstrated that play helps children to overcome their resistance to therapy. This is because, through play, children are not only expressing their feelings but also releasing stress by playing it out (Porter et al., 2009). The benefits of play in children's emotional wellness have given play the potential for therapeutic use.

Despite the proven benefits of play for children, there has been evidence showing that most formal education systems limit children's time spent playing. According to Gleave (2009), the academic stress children face increases as they get older. After children enter the formal education system, they will be facing commitment to homework. They not only need to spend more time in classes in school but also spend time on homework after school. This busy lifestyle reduces their free time to play. Zhao et al. (2015) have shown evidence of the negative influences of academic stress on the possibility of children developing mental health issues. The combination of decreased time in play, which can benefit children's mental wellness, and increased academic stress, which could damage children's mental wellness, potentially increasing children's emotional problems after they enter the formal education system. However, despite the increased emotional needs after entering the formal education system, people's expectations for a good teacher in the formal education system are focused on the teachers' teaching and the results of students' learning. None of the qualities reported was relevant to children's emotional needs (Liu and Meng, 2009). Social expectations of a good teacher in kindergarten in China are found to be related to children's social and emotional needs, such as love and respect for children (Cui et al., 2016). This may indicate that Chinese kindergarten teachers would be more aware of providing emotional support for the children than the teachers working in the formal education system.

Furthermore, Chaplin and Aldao (2013) suggested that children are more likely to show all their emotions in front of parents, especially negative emotions because children would expect their parents to accept these negative emotions. Thus, these findings may suggest that parents are important emotional support for children, especially after children enter primary school.

Lin and Bratton (2015) identified the low number of studies on the issue as one of the challenges in researching the effectiveness of play therapy. Furthermore, it was also identified that previous research on the effectiveness of play therapy has overlooked some key areas, such as the effectiveness of different types of play therapy and its effectiveness across different cultures (Lin and Bratton, 2015). This may indicate the lack of evidence on the effectiveness of play therapy.

Some research has shown the effectiveness of play therapy in alleviating the developmental or emotional difficulties children have faced. Money et al. (2021) have completed a systematic literature review that showed the promising effectiveness of play therapy on children with developmental difficulties. Cheng and Ray (2016) undertook research to test the effectiveness of child-centered group play therapy on children with social-emotional problems. Children with social-emotional problems were randomly assigned to the intervention group or the control group (Cheng and Ray, 2016). Based on the results of a pre-test and a post-test on the parents' and teachers' reflections on children's social-emotional abilities, the effectiveness of child-centered group play therapy has been proven (Cheng and Ray, 2016). Santacruz et al. (2006) undertook to test the effectiveness of play therapy on children's fear of the dark. The researchers designed a play therapy program, and the parents were trained to conduct the therapy at home with their children. Parents were asked to evaluate children's symptoms of their fear before and after the program. The results proved the effectiveness of play therapy in reducing children's symptoms of a fear of the dark.

From the research above, play therapy is not only effective for severe social-emotional disabilities; it can also be effective for reducing other common problems in children, such as children's fear of the dark.

The increased globalization in China may have reduced the cultural differences in Chinese perceptions of therapy for children. Thus, even though there has been a lack of evidence regarding Chinese parents' perceptions of play therapy, research on parents' perceptions of therapy for children in other cultures may have shown implications in common perceptions of Chinese parents and parents from other cultures to play therapy.

Hronis et al. (2020) tested the Western parent's perspectives on the factors that influence their decisions regarding their children's involvement in cognitive behavior therapy, and five themes were identified: the parents' ability to recognize their children's emotional states; their ability to take on the role of the therapist at home; therapists' knowledge and experiences; obstacles in the way of therapy (time and money restraints), and how cognitive behavioral therapy can be useful to children's needs. Other factors derived from previous research that have been identified as indicators of weather parents seek help from mental health services for children are: negative perceptions of mental health services (Wang et al., 2019); the level of the severeness of the children's problems (Merikangas et al., 2011); the ability to recognize children's problems (Sayal et al., 2006); living areas (urban or rural) (Morales et al., 2020); knowledge of therapy and the process of seeking help from mental health services (Reardon et al., 2017). These findings from previous research have facilitated some possible influences on Chinese parents' decisions regarding seeking professional help for their children's emotional needs.

Culture enormously influences children's ways of playing and people's understanding of play. It is widely believed that this happens in many ways: “Each culture sees play in a distinct way, and the reaction of adults to play also varies” (Gosso, 2009, p. 80). Culture not only influences people's attitudes toward play, but also influences the ways children play. Jones (2020) has suggested that children would play out incidents that they have seen in their life. For example, if children had seen an incident of a men working on lights that fell off a building, their play would reflect on the incident. According to Sutton-Smith (2009), when children play to reflect on events they have seen around them, they bring adult culture into their play. At the same time, children would add their understanding of the events into the play too. This is probably one of the reasons why play is defined as an effect of culture by Carvalho and Rubiano (2004).

China has a very special cultural context for parenting styles. Lily et al. (2022) suggested that the way in which children are raised is greatly influenced by culture, and traditional values and practices rooted in Confucianism continue to exert a significant impact on child-rearing practices in China. According to Wang (2004), familial piety is emphasized in Confucian thinking. In Chinese culture, parental control over children is likely to be emphasized in the parenting style. Thus, it is implied that Chinese parents' attitudes toward therapy can significantly influence the children's opportunities of being involved with therapy. Furthermore, Shi et al. (2020) have demonstrated that most Chinese people are reluctant to go to mental health services because of the stigma attached to having a mental illness. Parental resistance to therapy with children also seems to be rooted in Chinese culture. According to Shen et al. (2018), Chinese parents are likely to feel guilty about their children's psychological problems and see it as their failure in child-rearing, so they would rather try to solve the problems themselves rather than seek outside assistance.

Even though playing has been recognized to be very important to children's learning and growth by many scholars (Russ, 2004; Ginsburg, 2007), play is still valued less in Chinese culture than in Western cultures (Kao, 2005). In China, the value of play is downplayed by many parents and educators. According to Cao (2000), in Chinese culture, play is often valued as a means of entertainment, but other values of play are less likely to be acknowledged in Chinese culture: “Oftentimes, Chinese parents allow very young children to indulge in play. However, once their formal education starts, parents tend to discourage play and focus more on children's academic performance” (Shen, 2016, p. 332). Due to the cultural influence in China, it is possible for parents to discredit the value of play and discourage their children to participate in therapeutic play. For example, “in a play therapy intervention program for earthquake victims, a boy withdrew because his parents discouraged him from participating” (Shen et al., 2018, p. 330). This illustrates the tension between children's access and the way in which parental power can facilitate or limit access based on their attitude.

It's worth noting that “[since] the advent of the open-door policy, China has undergone dramatic socioeconomic changes and increasing globalization, which has brought more opportunities for contact with diverse ideologies about childrearing and education from other parts of the world” (Lin et al., 2018, p. 84). While the Chinese government endorses play for children by initiating early years educational reforms in China, some parents have changed their perspectives on child-rearing (Lin et al., 2018). Thus, it is critical to understand Chinese parents' attitudes toward this culturally fluid phase of play therapy.

In conclusion, with the academic stress that comes from formal education increasing with the children's age, and parents' lack of time to interact with their children to form healthy parental relationships, more and more people are concerned about children's mental wellbeing in China. With the increasing need for emotional support services for children, evidence has shown that some Chinese parents are reluctant to get help from these services. Play therapy as a therapy for children has been proven to be effective in previous research. Even though therapy may not be a familiar term for parents in China, play is something all parents are familiar with. Thus, the aim of this research is to learn about the barriers of Chinese parents who are reluctant to become involved with psychotherapy through an exploration of their perceptions of play therapy and other related factors. The main research questions for this research are:

1. What are chinese parents' and teachers' perceptions on the role of play and how may this connect to the parents' decisions regarding letting their children engage with play therapy?
2. What are the chinese parents' and teachers' perceptions about children's emotional needs and how it may be related to parents' decisions regarding letting their children engage with play therapy?
3. What are chinese parents' and teachers' perceptions of therapy and play therapy and how may they relate to parents' decisions regarding letting their children engage with play therapy?

## 2. Methodology

### 2.1. Data collection

The method used in this research is a mixed method of qualitative research and quantitative research. The quantitative data was used to facilitate the qualitative data. Quantitative data were collected first by using a questionnaire to provide a general description of the parents' perceptions of children's emotional needs and therapy. Then, qualitative data was collected from three participants, who filled out the questionnaire. By asking open-ended questions, more details of the factors in each case that may relate to the participants' opinion were explored. Finally, a focus group interview was conducted with three teachers as participants to provide a different perspective on the same issue. By using mixed methods, the results would provide a comprehensive answer to the research questions.

The questionnaire was used to explore the parents' perceptions of children's emotional needs and their perceptions of therapy. For example, parents were asked to rank the extent to which they agreed with the statements related to therapy. The questions in the questionnaire were based on the research and theories presented in the literature review chapter. For example, one of the questions in the questionnaire is: “Do you agree children in the scenario below should be engaged with therapy? Choose from 1 to 5 (1. Strongly agree 2. Agree 3. Somewhat agree. 4. Disagree 5. Strongly disagree).” The scenarios included “the child is afraid of the dark,” based on the findings of the research undertaken by Santacruz et al. (2006), which was introduced in the Introduction chapter.

Secondly, after collecting data through the questionnaire, one-to-one semi-structured interviews conducted online were used to explore parents' perceptions of play, children's emotional needs, play therapy, and factors affecting their decisions regarding allowing their children to engage with play therapy. Questions in the interview were designed based on the previous research presented in the literature review. For example, to explore factors that influence parents' decision-making, one of the questions in the interview was “How much do you think it would cost for play therapy?” The rationale for this question was based on the research undertaken by Hronis et al. (2020), which was introduced in the Introduction Chapter.

Finally, a focus group was organized to explore teachers' perceptions of play, play therapy, teachers' awareness of children's emotional needs, and teachers' perspectives on factors parents would consider when deciding on their children's engagement in play therapy. The focus group was conducted through an online voice call. The questions asked in the focus group are selected from the questions asked in the interview. Since the themes explored through the questions are the same, but the total time for each participant to respond in a focus group is less than the time in one-on-one interviews, fewer questions were asked in the focus group interview.

All research methods were piloted with a parent and a teacher who are not participants in this research. Generally, the feedback was positive, however, the format of the last two questions in the questionnaire was reported as hard to understand. The questions were edited after the piloting.

### 2.2. Sampling

Sixteen parents of children aged from three to fourteen finished the questionnaire. Three participants participated in the online one-to-one semi-structured interview: a mother of a five-year-old boy (Parent A), a father of a seven-year-old girl (Parent B) and a mother of a twelve-year-old boy (Parent C). The three children are in different age groups. The five-year-old boy is in kindergarten, where he was identified with social emotional difficulties. The seven-year-old girl just started her first year in primary school. The 12-year-old boy is in the sixth grade of primary school and will enter middle school soon. Both Parent B and Parent C reported that their children have no social-emotional problems. Three teachers participated in the focus group. There is a hierarchy classification of the cities in China based on the level of economic development. Teacher B is working in a kindergarten in a tier 1 city, which is a highly developed city. Teacher A is working in a kindergarten in a tier 2 city, which is ranked below the tier 1 cities. Teacher C is working in a kindergarten in a rural area.

This research used a snowball sampling technique to recruit the participants. More specifically, the researcher used personal friendship networks to recruit the participants. According to Handcock and Gile (2011), the participants recruited through snowball sampling are not meant to be representative. Similarly, the participants recruited for this study are not meant to be representative of the larger population. Taherdoost (2016) suggested that one of the benefits of snowball sampling is the convenience when recruiting the participants. Because of the current situation of COVID-19, it is difficult to obtain access to settings where there is no personal connection, so snowball sampling has been useful. It enabled me the access to recruit participants.

### 2.3. Ethical considerations

Ethical issues are an essential consideration for researchers, especially when conducting collaborative research. Research ethics have often been regulated by the organizations researchers work in. If the researchers refuse to include ethical considerations in their research, not only will peers doubt the value of the research but the participants who have contributed to the research and the readings who might be impacted by the research would be let down (Mauthner et al., 2012). This dissertation is a collaborative research study. It involves the cooperation between the researcher and children's parents and practitioners in China. This research has been given ethical approval by the internal ethics approval authority of the UCL Institution of Education. One of the questions that should be considered for collaborative research is how to make sure that researchers are undertaking studies with others instead of undertaking studies *on* others (Fernandez et. al., 2022). In other words, it is important to make sure the participants are participating in the research voluntarily, instead of being coerced into the research. There are three main ethical aspects that should be considered in this research.

#### 2.3.1. Informed consent

For this research, the participants received an information sheet that gave them information about the research and what to expect if they participate in the study. They made their own decision regarding their participation after reading the information sheet. They were asked to sign a consent form if they decided to participate in the study. Before the questionnaire, interview, and focus group, the researcher asked the participant again to make sure they understood everything on the information sheet and consent form to make sure they participated in the research voluntarily.

#### 2.3.2. Avoidance of harm

According to the National Institutes of Health (2018), researchers should minimize the potential harm to the people who are involved in research. In this study, there are two potential dangers. Firstly, it may cause emotional disturbance for the parents of children in the special education program when talking about the reasons behind sending their children to the special education program. In order to minimize the harm, participants were informed that they could stop participating in the research anytime they wanted, and they were informed again before the interview. A link to a local emotional support service was provided on the information sheet. Secondly, for the teachers who would be participating in the focus group, there is a potential risk to their career if their statements about their work were disclosed. To minimize this risk, all their statements in the focus group discussion will be kept confidential, and there will be a statement on the consent form about asking the teachers to keep each other's statements in the discussion confidential.

#### 2.3.3. Confidentiality

Based on a definition made by the National Institutes of Health (2018), the purpose of confidentiality is to safeguard research participants from potential harm, such as stress, embarrassment, or unwanted publicity that may arise from the dissemination of research outcomes. To achieve this, participants were guaranteed that the information collected through the research will remain anonymous and will only be used for academic purposes.

#### 2.3.4. Validity

According to Oluwatayo (2012), validity and reliability are the two most important aspects of measuring tools in research. Validity has been defined as the degree of accuracy of the measuring instrument that is used in research (McBurney and White, 2007). In other words, validity involves issues related as to whether the measuring tools used in the research are relevant to the research question. In this research, three aspects related to validity are involved: face validity, content validity, and triangulation validity.

Face validity addresses the question: do questions used in the measuring instrument “appear to be relevant, reasonable, unambiguous and clear?” (Oluwatayo, 2012, p. 392). In order to achieve face validity, the measuring instruments for this research have been reviewed by an expert in the field (my supervisor, Dr Jones). Content validity addresses the issue of “the extent to which the instrument of measurement shows evidence of fairly and comprehensive coverage of the domain of items that it purports to cover” (Oluwatayo, 2012, p. 393). The instruments of measurement in this research are the questions designed for the questionnaire, interview, and focus group. This research achieved content validity by clearly identifying the purpose of the questions, and the questions were based on the findings from the literature review.

According to Teddlie and Tashakkori (2021), triangulation validity refers to collecting data from various resources to develop a deeper understanding of the issue related to the research question. To achieve triangulation validity, this research collected data by using three different tools: interviews, questionnaires, and focus groups. Furthermore, the research included results from two different perspectives on the same issue—teachers' perspectives and parents' perspectives, to achieve validity through triangulation. One of the shortcomings of the triangulation approach is that when the data collected through different tools conflicts with each other, it may be difficult to find a clear answer to the research questions (Abdalla et al., 2018). However, even when the data is conflicting, the whole picture of the issue being researched is still enriched by the data. The data collected through triangulation can show the complexity of the issue.

### 2.4. Approaches to data analysis

This research used the thematic analysis method to analyze the qualitative data. Firstly, the recordings of the interviews and the focus group were reviewed, and the keywords relevant to the research questions were noted. Then, by comparing the key terms, common themes were identified. The researcher then reviewed the relationships between the themes and the key works to draw thematic maps. Finally, the themes were refined, and the relationship between the keywords and the refined themes was presented by the finalized thematic map. According to Siedlecki (2020), in descriptive designs that use a questionnaire, the quantitative data can be reported as percentages. Thus, in this research, the quantitative data collected through the questionnaire was analyzed by calculating the percentages of the participants selected for each choice under each question. Since the aim of collecting the quantitative data in this research is to describe the samples' opinions, only a simple descriptive analysis would be necessary. By comparing the proportion of the participants under each choice, the perception most people agreed with and disagreed with provides a wider picture of the sixteen parents' perceptions of children's emotional needs and therapy.

## 3. Results

The findings from each data collection method are structured by the criteria explored through each research method. There are two criteria explored in the findings from the questionnaire: awareness of children's emotional needs and parents' perceptions of therapy. There are four criteria (themes) explored from parents' perspective by using interviews: perception of play, awareness of children's emotional needs, parents' perceptions of play therapy, and factors parents would consider for participating in play therapy. The same four criteria (themes) are explored from teachers' perspectives by using a focus group interview. The relationship of each criterion with each research question is presented in Table 1.

**Table 1:** Research questions and relevant criteria/themes explored.

**Research question**	**Relevant criteria (theme)**
What are Chinese parents' and teachers' perceptions on the role of play, and how may this connect to the parents' decisions on letting their children engage with play therapy?	Perceptions of play
	Factors related to decisions of children's engagement with play therapy parents would consider
What are Chinese parents' and teachers' perceptions on children's emotional needs and how it may be related to parents' decisions on letting their children engage with play therapy?	Awareness of children's emotional need
	Factors related to decisions of children's engagement with play therapy parents would consider
What are Chinese parents' and teachers' perceptions of therapy and play therapy, and how might this be related to parents' decisions on letting their children engage with play therapy?	Perceptions of therapy and play therapy
	Factors related to decisions of children's engagement with play therapy parents would consider

### 3.1. Participants

Seventeen participants responded to the questionnaire but only sixteen finished the questionnaire. The data of the participant who did not finish were excluded from the results. Among the sample of sixteen parents, eight are parents of boys and eight are parents of girls. The age of the participants' children ranged from three to fourteen with a mean value of 7.44. Children are classified into different age groups based on the age group classification in the Chinese education system. Five children aged from three to six are identified as children in the early years of education. Within the five children, there are four girls and one boy, and the boy is the only child within the sample (n=16) who is identified with social-emotional difficulties by his parent. Among the ten children aged from seven to twelve who are identified as children of primary school age, seven are boys and three are girls. One child aged from thirteen to fifteen is identified as middle school age.

Three participants participated in the interview. They are parents with children of different ages and different genders. These differences may have influenced the parents' perspectives on the four main themes, which will be discussed in the next section.

As was mentioned in the sampling section, the three participants in the focus group work in different settings. This difference may have been related to the difference in the teachers' perceptions, which will be discussed in the next chapter.

### 3.2. Main findings

The findings of this research have created insights into Chinese parents' and teachers' awareness of children's emotional needs, and parents' perceptions of play, therapy, and play therapy. Factors that parents may consider when deciding on letting their children be engaged in play therapy are also explored in interviews and focus groups.

#### 3.2.1. Theme 1: perception of play

##### 3.2.1.1. Subtheme: time and materials of play

Regarding the time children spend on playing, both Parent A and Parent C reported their children's time spent on the play was more than 2 h every day. For example, Parent C said: “…playing time for him during school days is 2–3 and 5–6 h every day during vacations…” It is worth noting that Parent B reported playing time after school was only up to 30 mins. He went on and indicated schoolwork as influencing the time children spend on play: Parent B: ‘Now she's entered primary school, she doesn't spend much time playing… Only up to 30 mins every day.' This may have indicated that schoolwork caused a reduction of time spent on play, which in turn caused a reduction of materials children play with. Regarding the materials children play with, both Parent A and Parent B commented on avoiding electronic devices. Parent C, on the other hand, reported that video games and mobile games are important for the child's daily play. Parent C also indicated the variety of materials their child plays with was reduced due to schoolwork. This can be seen from the examples in Supplementary Table 1.

##### 3.2.1.2. Subtheme: who children play with

All three participants in the interviews reported that children spend less time playing with peers after school and playing with peers is not part of children's daily activity after school. Some occasions when children play with peers are related to these subthemes: “outdoor play,” “at school,” “on weekends.” Both Parent A and Parent C focused on who the child plays with after school. Parent A reported that occasionally, when she had time to take the child outside, the child would play with peers. Parent C responded that the child would play with peers on weekends. Parent B indicated the child played with peers at school, not after school, and the possible reason was that peers live far away. Further subthemes and specific examples can be seen in Supplementary Table 2.

Regarding whether children play with their parents, Parent A reported she barely plays with the child. Parent C reported the child plays with the father all the time. Both Parent C and Parent A indicated that parents busy with work influences whether parents play with children. Parent A mentioned her job takes up too much of the time, in turn limiting the extent to which she can spend time with her child. Parent C mentioned how she and her husband did something different to other parents they know. They quit their jobs, so they could have more time to spend with their children. Parent B, on the other hand, addressed the time families in general spent playing with their child. He reported other families sometimes play with the child, but for less time than before. The reason given is that children preferred playing alone.

Regarding the teachers' perception of play, the main finding is that the children in kindergarten normally would spend time playing outdoors and indoors. Most of the time was spent on free play with peers. Further subthemes and specific examples can be seen in Supplementary Table 3.

##### 3.2.1.3. Importance of play

**Figure 1:**
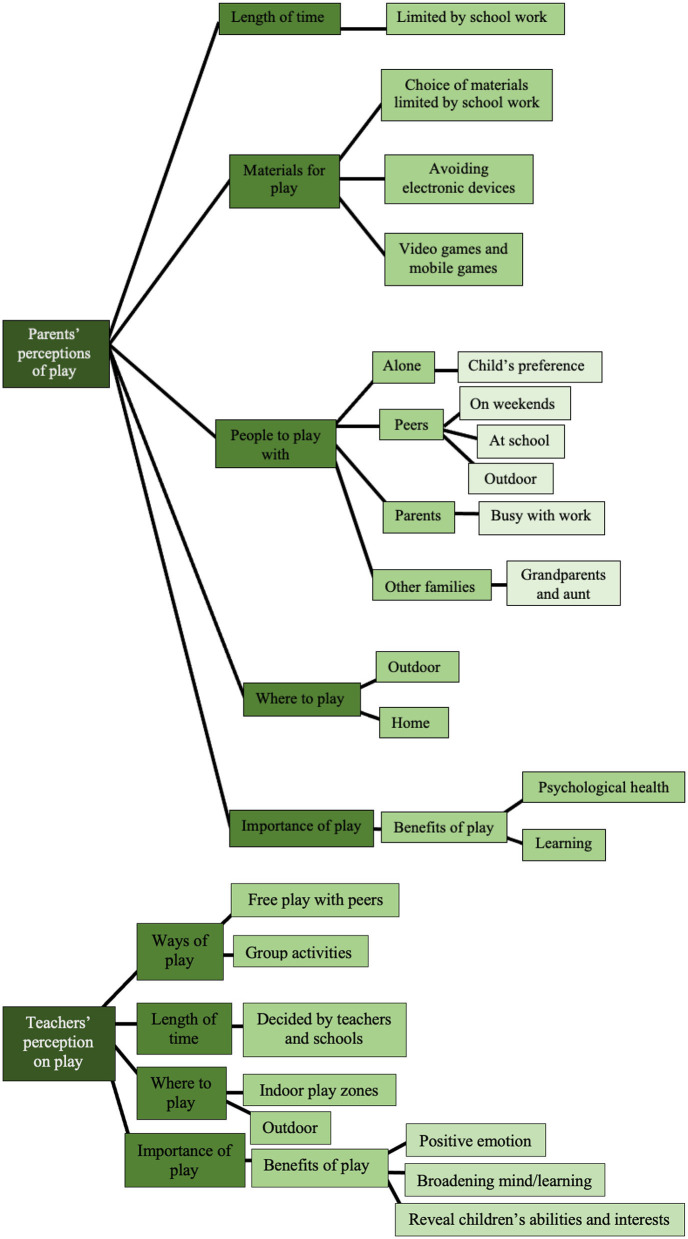
Subthemes and further subthemes for theme 1: perception on play.

All three parents reported play being important for children due to the benefits play can bring to children's learning, and psychological health. Like the three parents' perceptions of play, the three teachers also believed play is important for children. The reasons why play is important are also related to the benefits of play for children: “positive emotions,” “broadening the mind/learning.” Furthermore, Teacher A reflected that the trait can “show children's abilities and interests” under the subtheme “importance of play.” She further suggested how this trait can benefit teachers in designing the activities and evaluating children's abilities. Further subthemes and specific examples can be seen in Supplementary Tables 4, 5. The summary of subthemes and further subthemes derived from the theme of perceptions on play that appeared from the responses of the parents and teachers is presented in Figure 1.

#### 3.2.2. Theme 2: awareness of children's emotional needs

As shown in Table 2, in the questionnaire, all sixteen participants reported that they believe children have emotional needs. All sixteen participants rated the difficulties of interpreting the five emotions of their children as very easy, easy or medium; no participant rated any emotions of their children as hard or very hard to read. This indicates that the parents think they can interpret children's emotions well.

**Table 2:** The number of participants responded to the level of difficulties interpreting their children's emotions.

	**Very easy**	**Easy**	**Medium**	**Hard**	**Very hard**
Sad	12	3	1	0	0
Angry	14	1	1	0	0
Happy	14	2	0	0	0
Anxious	6	4	6	0	0
Scared	10	4	2	0	0

Within the sample size, anxiety was rated as relatively hard to interpret when expressed by children. Anger and happiness were rated as relatively easy to interpret from children, with more participants thinking that happiness was “very easy” and “easy” to interpret.

##### 3.2.2.1. Children's styles of expression

As presented in Table 3, most parents among the sample size strongly agreed or agreed that their children express feelings through behaviors. Fewer parents strongly agreed or agreed that their children express their feelings through verbal expression. Most parents among the sample disagree and strongly disagree that their children rarely express feelings. The data collected from the interviews supported the findings in the questionnaire. All three parents reported they were able to interpret their children's emotions. Interpretation methods were through observations of children's facial expressions, behavior and verbal expressions (Specific examples can be seen in Supplementary Table 6). The three teachers in the focus group also reflected on the same traits in their ways of interpreting children's emotions (Specific examples can be seen in Supplementary Table 7).

**Table 3:** Parents' attitudes toward the statements on their children's ways of expressing emotions.

	**Strongly agree**	**Agree**	**Not sure**	**Disagree**	**Strongly disagree**
My child directly expresses his/her feelings	5	6	2	1	0
My child express feelings through behaviors	7	8	1	0	0
My child rarely expresses feelings	2	0	2	9	1

**Figure 2:**
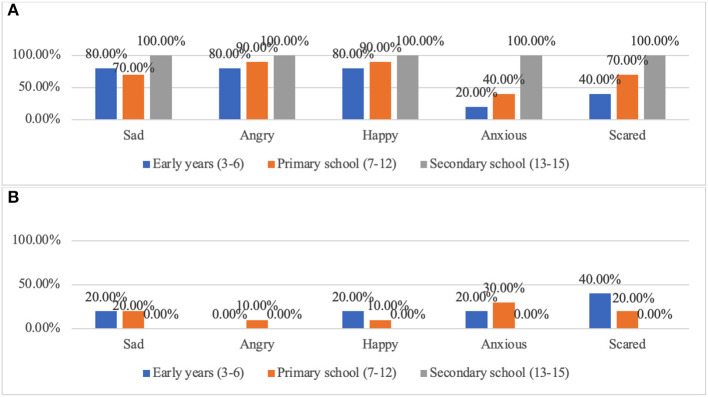
Parents of children in different age groups reported the level of difficulty in interpreting their children's emotions as very easy and easy. **(A)** Very easy-age group. **(B)** Easy-age group.

The association of children's ages and genders and the difficulties for parents in interpreting their emotions were further explored in the questionnaire. Based on the percentages of the sixteen parents' ratings, emotions of children of secondary school age are the easiest for the parent to interpret. The proportion of parents with children of primary school age who rated angry, happy, anxious, and scared are very easy and easy (Figure 2) to interpret are higher than the proportion of parents with children in early years. The proportion of parents with children in early years age who rated sad as easy or very easy to read is 10% higher than the proportion of parents with children of secondary school age.

**Figure 3:**
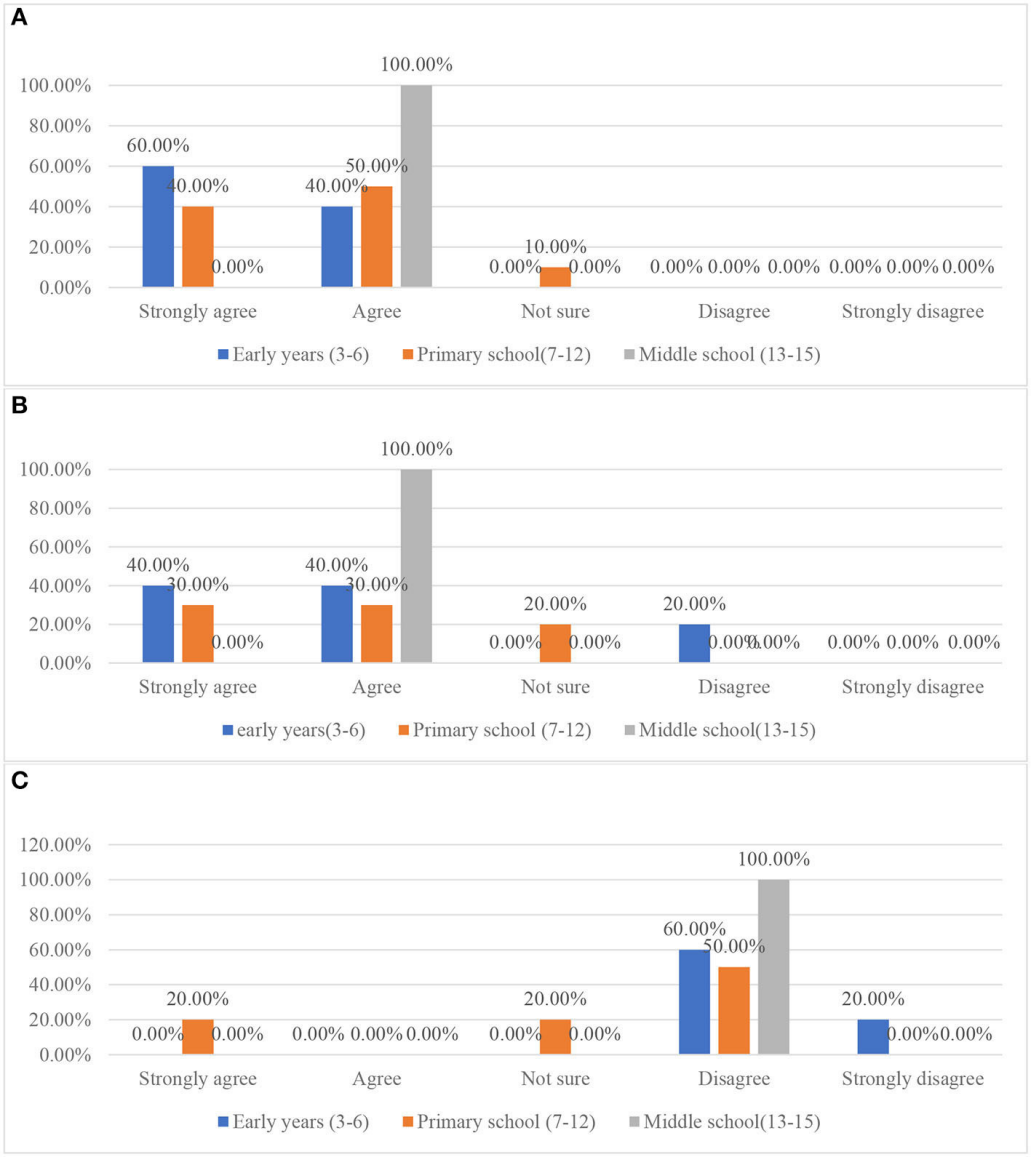
The attitude of parents of children in different age groups to the statement: my child expresses feelings through verbal expression, behavior, or my child rarely expresses feelings. **(A)** Children express feelings through behavior. **(B)** Children express feelings through verbal expression. **(C)** Children rarely express feelings.

In Figure 3, regarding the possible influence of children's ages on their ways of expressing emotions from parents' perspectives, the proportion of the parents of children of middle school age reported that children's expressiveness through verbal expression and behavior is the highest among the three age groups. The proportion of parents of early years children who reported that their child expresses feelings verbally and behaviorally is higher than the equivalent proportion for parents of children in primary school. The proportion of parents of children in primary school who reported their child rarely expresses feelings is higher than the parents of children in other age groups.

**Figure 4:**
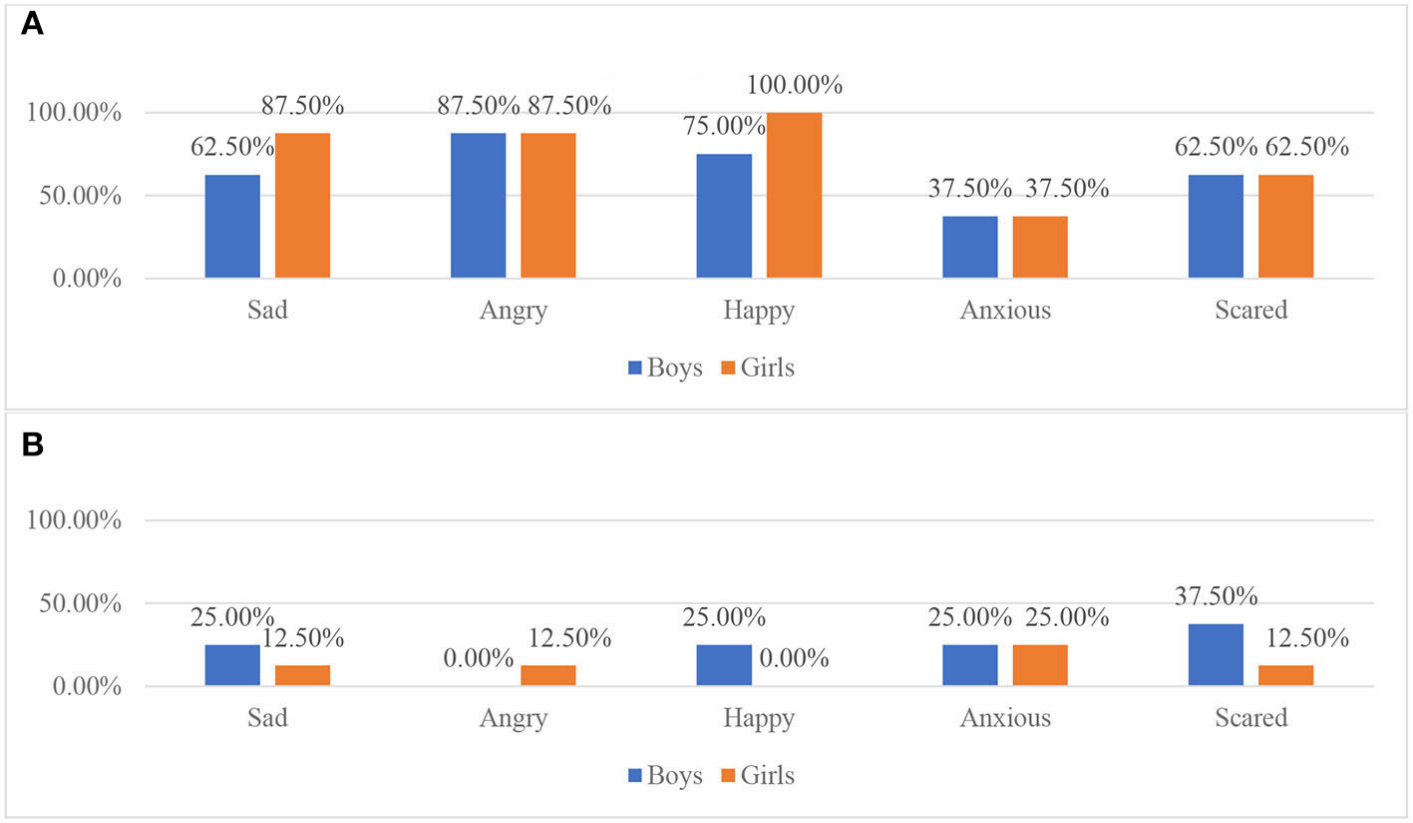
Parents of children of different gender reported the level of difficulty in interpreting their children's emotions as very easy and easy. **(A)** Very easy-gender. **(B)** Easy-gender.

In terms of the influence of gender on the difficulties parents' experienced when interpreting a child's emotions, the proportion of parents of girls who rated sadness and anger as very easy and easy (Figure 4) to interpret is higher than the proportion of parents of boys. The total proportion of parents of boys who rated happiness and anxiety are the same as the proportion of parents of girls. However, there is a higher proportion of parents of girls who rated happiness as very easy than the proportion of the boys, which may indicate the parents think it is easier to interpret happiness in girls than in boys. The proportions of boys' parents who rated fear as very easy and easy to interpret is higher than the proportion of girls' parents. It has been indicated that within the sample size, from the parents' perspective, most girls' emotions (sadness, anger, happiness) are easier to interpret than the boys' emotions.

**Figure 5:**
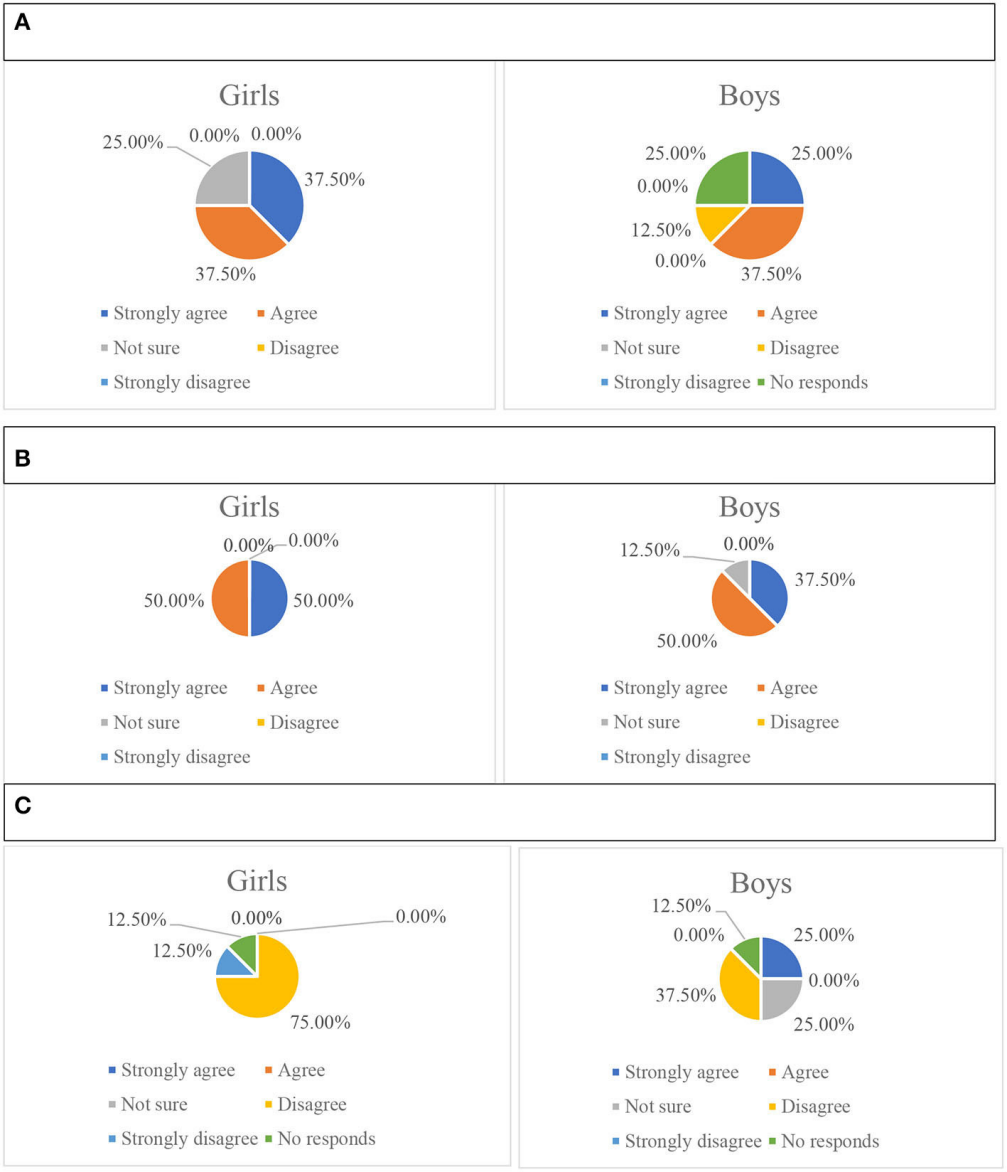
The attitudes of parents of children with different gender to the statements. **(A)** My child expresses feelings through verbal expression. **(B)** My child expresses feelings through behavior. **(C)** My child rarely expresses feelings.

In Figure 5, relating to the gender difference of children and their ways of expressing emotion, a higher proportion of girls' parents strongly agreed and agreed to their children expressing feelings through verbal expression and behaviors than the proportion of boys' parents. A higher proportion of boys' parents strongly agreed and agreed that their children rarely express feelings.

##### 3.2.2.2. Possible cause of hard feelings for children

In the interviews, Parent A, whose child was identified as having social emotional problems, suggested the child may experience complex feelings in social situations. The other two parents who identified their children with no social-emotional problems reflected on parents' negative influences on children's emotional states. Specific examples can be seen in Supplementary Table 8.

##### 3.2.2.3. Important emotional support

**Figure 6:**
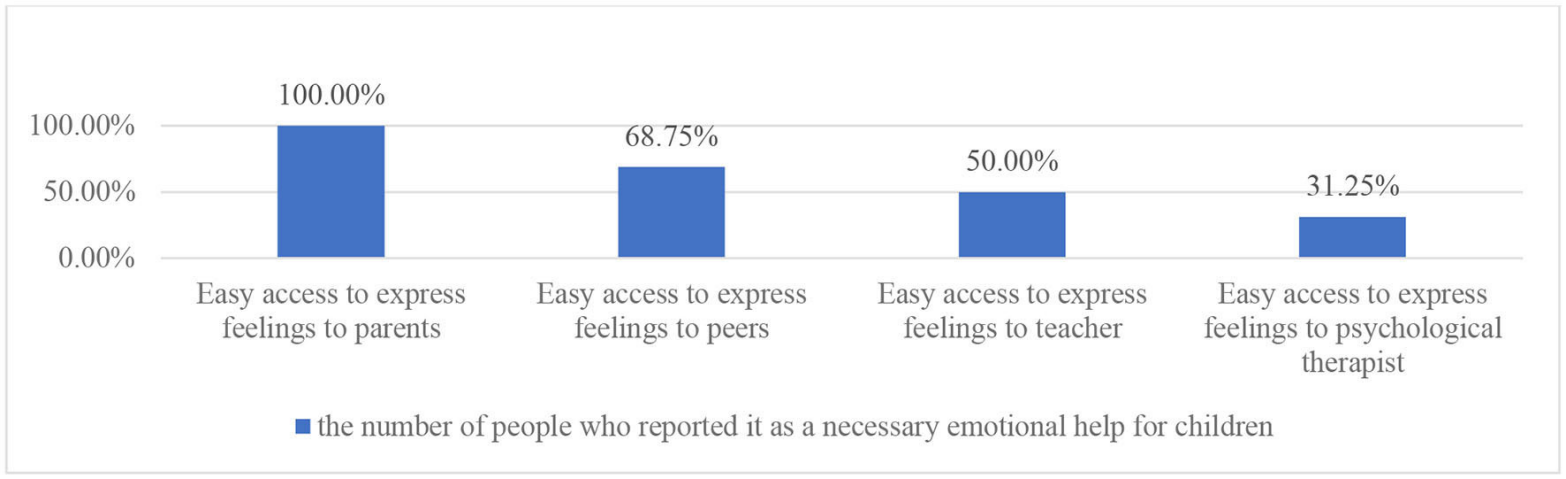
Participants' responses regarding important emotional support for children.

In Figure 6, Regarding the topic of emotional support for children, there was a clear difference between different elements of the questionnaire. All the parents believe that children talking about feelings with parents is important, with peers and teachers being 68.75 and 50.00% respectively. The smallest proportion of parents, 31.25% of the sample, reported that expressing feelings with a therapist is important for children. The data collected through interviews is similar to the findings in the questionnaire.

In the interviews, all three parents reflected on the importance of parents being children's emotional support. This may also support what has been suggested before on how some parents in China think children's emotional needs are part of their responsibility as parents. Only Parent A reflected on how teachers may have supported her child emotionally. None of the parents in the interviews think peers are important emotional support for children. Specific examples can be seen in Supplementary Table 9.

In the focus group, the three teachers suggested parents, teachers and peers are all important sources of emotional support, with different ranking levels. The parents were ranked first, then the teachers, and finally the peers. Specific examples can be seen in Supplementary Table 10. The three teachers further discussed the ways early years teachers would support children when they are showing negative emotions. They would communicate with the child and ask other peers to play with the child.

**Figure 7:**
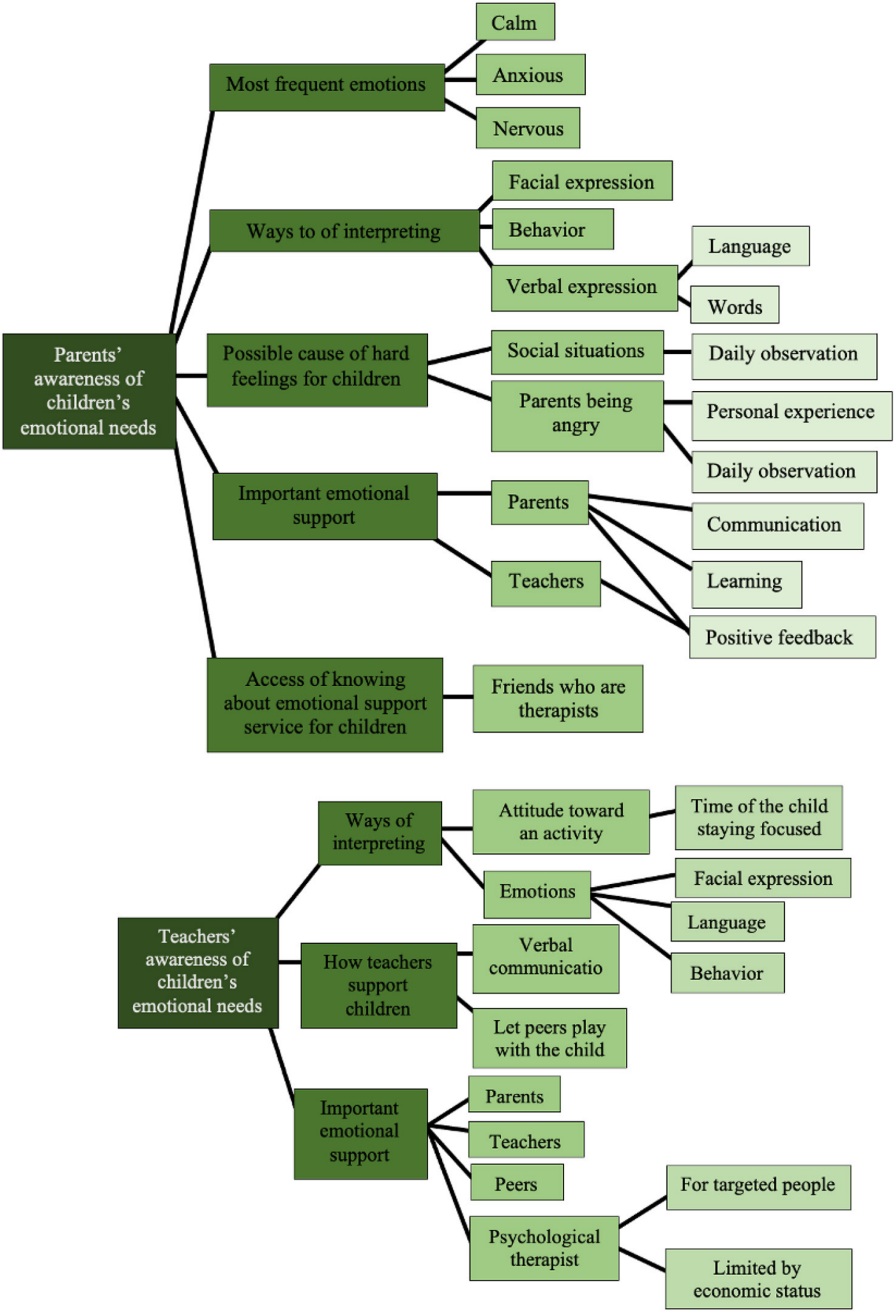
Subthemes and further subthemes for theme 2: Awareness of children's emotional needs.

The summary of subthemes and further subthemes derived from the theme of awareness of children's emotional needs that appeared from the responses of the parents and teachers are presented in Figure 7.

#### 3.2.3. Theme 3: perceptions of therapy and play therapy

##### 3.2.3.1. Subtheme: perceptions of therapy

In the questionnaire, fifteen participants reported that they had heard of therapy and one participant reported not sure. Six participants reported knowing what therapy is; three participants did not know, and seven participants reported not sure. In the interview, the parents interpreted this finding was due to the lack of publicity of therapy in China. The three parents indicated that people in China have very limited access to information about therapy. Specific examples and further subthemes can be seen in Supplementary Table 11. Another trait of therapy that was reflected by the three parents in the interviews was that it is “only for people with disorders.” Other traits reflected by the three parents in the interviews are “not sure about the effectiveness,” “doubts on the qualifications of the therapist.” Specific examples and further subthemes can be seen in Supplementary Table 12.

**Figure 8:**
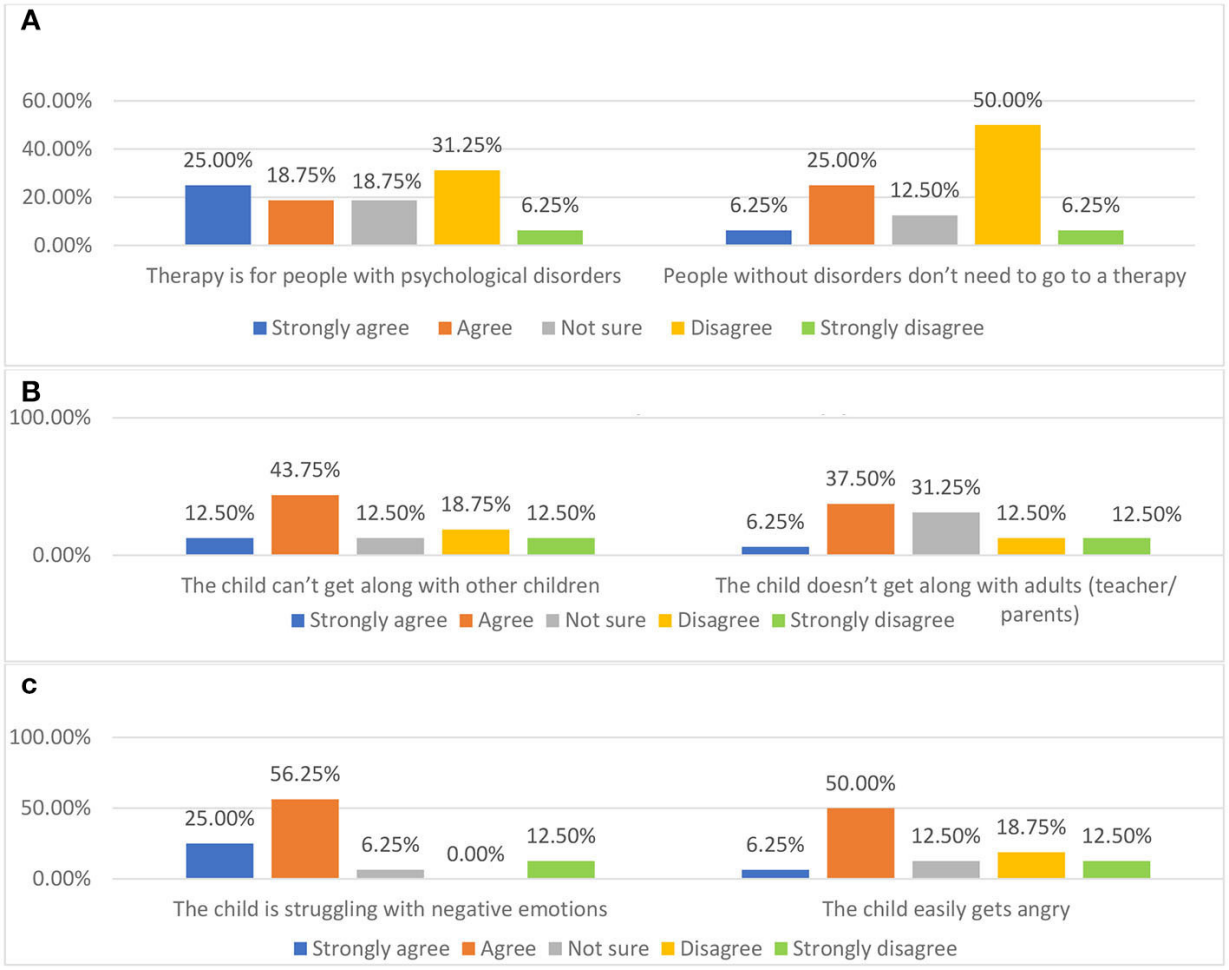
Parents' attitude to the statements related to therapy. **(A)** Parents' attitude toward therapy. **(B, C)** Situations in which a child may need therapy.

As shown in Figure 8, in alignment with the interviews, the data collected from questionnaire showed that most of the participants think therapy is for people with disorders. These findings may have proved the interpretation on why the fewest parents in the questionnaire sample think therapist is important emotional support for children stated above.

Regarding the circumstances children may need to be engaged with therapy, the results in the questionnaire are similar to the findings on parents' perceptions about therapy. As shown in Figure 8, most of participants think children who have social or emotional problems should be engaged with therapy. Most of the participants in the questionnaire were not sure about children needing therapy when they are afraid of the dark. Compared with the participants who reported agreed or strongly agreed, more participants disagreed or strongly disagreed with the statement.

##### 3.2.3.2. Perception of play therapy

Regarding parents' perceptions of play therapy, in the interviews, both Parent B and Parent C also suggested that only when children are identified with social-emotional disabilities would a professional therapist potentially be needed. Specific examples and further subthemes can be seen in Supplementary Table 13.

The teachers in the focus group interview had a similar perception. They think children who act very differently to other children and children with special needs should be involved with play therapy conducted by a therapist. For example, teacher A said, “for children who have special needs, it might be hard for them to be involved in the ways children normally play…play therapy is more likely to be needed for children with special needs.” This finding is understandable since play therapy is a kind of therapy, so they had similar perceptions on who needs therapy and who needs play therapy.

Interestingly, in the interviews, Parent B and Parent C also think play therapy can be a daily activity for any child to prevent future mental health issues. Specific examples and further subthemes can be seen in Supplementary Table 14.

Furthermore, Parent C suggested a parent may be able to conduct play therapy without any training if the parent knows the child well enough to understand the child's emotional needs (Supplementary Table 13).

##### 3.2.3.3. Perception on the cost of therapy/play therapy

**Figure 9:**
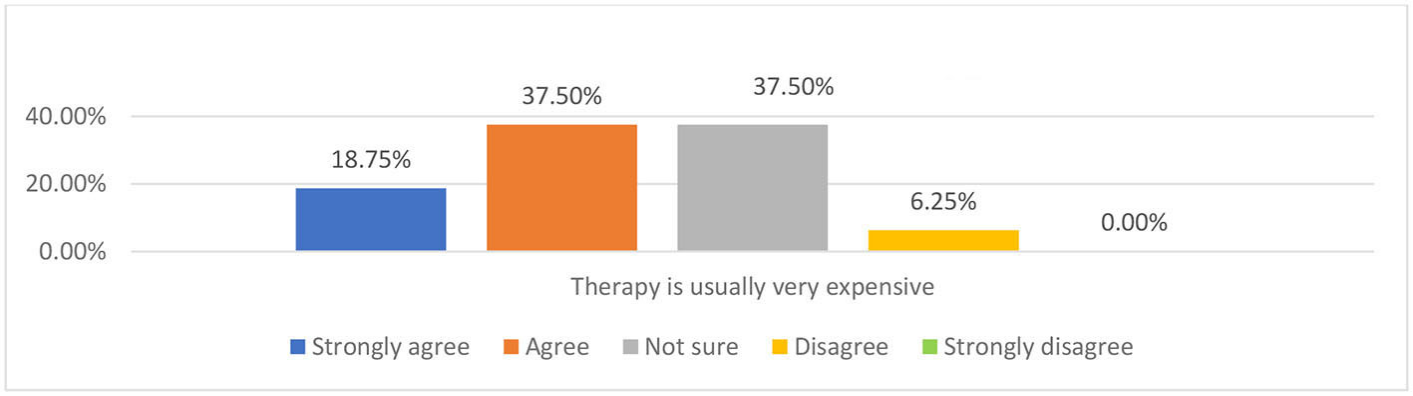
Parents' attitude to the statement “therapy is usually very expensive”.

As presented in Figure 9, in the questionnaire, most participants strongly agreed and agreed with the perception of therapy being very expensive. The other parents in the interviews and all three teachers in the focus group also think any therapy involving professional therapists is expensive. For example, Teacher C suggested that “normally, the parents wouldn't try to engage with therapy because the cost of it is so high.” In the interview, Parent B suggested he would spend around 30 pounds in total for play therapy. He said “my child is healthy, so I'd rather use the money to buy her some new clothes… Maybe, maybe around a hundred RMB (around $15) to let my child have the chance to be involved in such a novel experience.” However, his perception of the cost of therapy conducted by a therapist in China is that it is very expensive. This may have indicated that the cost of the therapy could be another factor limiting Chinese parents' access to seek help from mental health services.

All three parents in the interview think play therapy could be effective for children with social-emotional problems, such as “building up the child's confidence” and “managing emotional problems.” For example, Parent B said: ‘Through activities related to play, it can help children to overcome some difficult emotions, such as anxiety, depression etc. … I think play therapy is helpful.'

**Figure 10:**
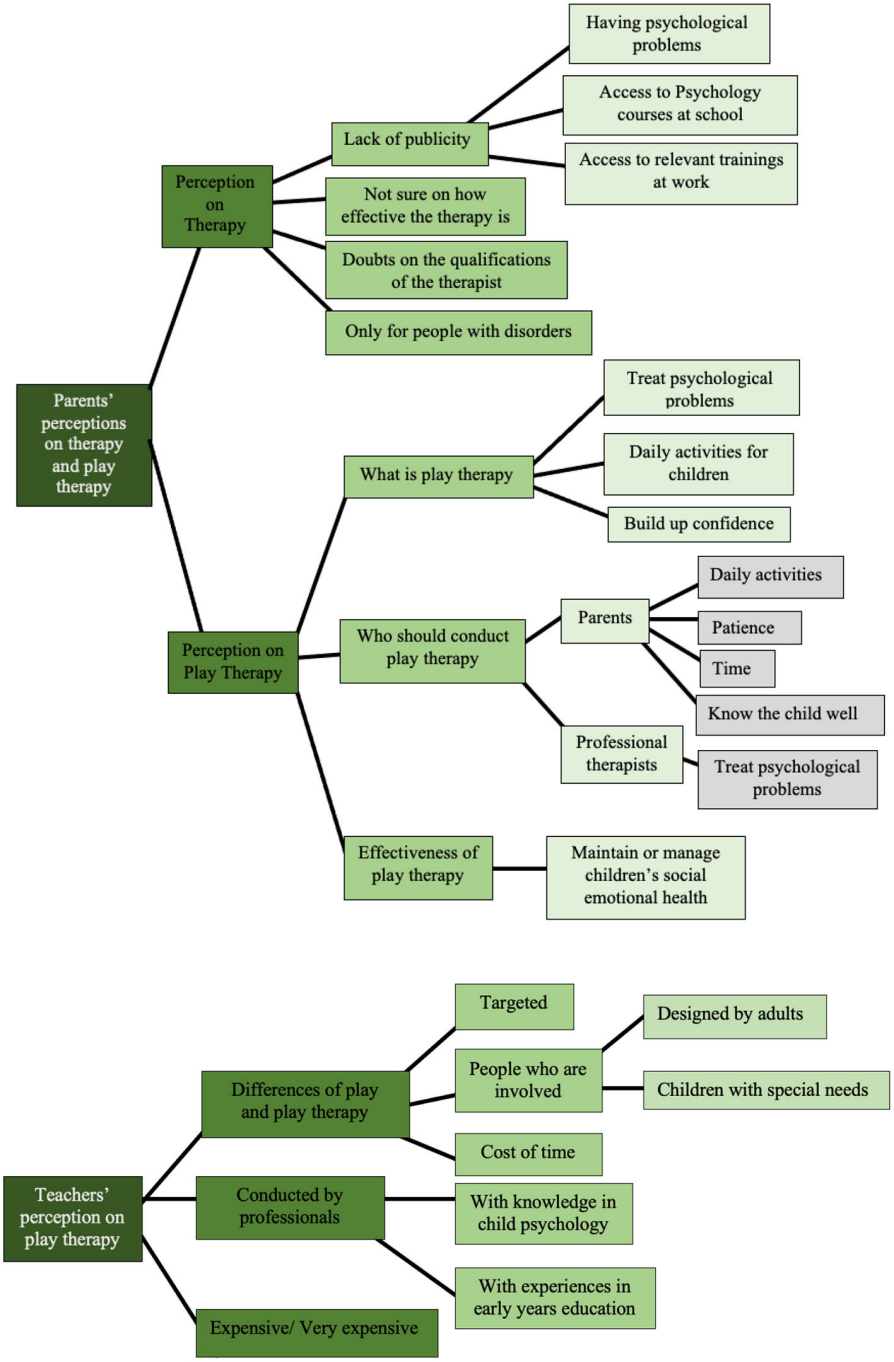
Subthemes and further subthemes for theme 3: perception on therapy and play therapy.

The summary of subthemes and further subthemes derived from the theme of perceptions on therapy and play therapy that appeared from the responses of the parents and the teachers are presented in Figure 10.

#### 3.2.4. Theme 4: factors influencing parents' decisions about engaging with play therapy

When talking about making decisions on children's engagement with play therapy, six subthemes were derived from the parents' perspective: “child's attitude toward therapy,” “access to the therapy,” “general rationale behind the therapy,” “whether the child needs therapy,” “effectiveness” and “cost.” The only common trait is “the child's attitude toward therapy.” Both Parent A and Parent B mentioned they would consider the child's reaction toward the therapy or the therapist. Parent A said “from a parent's perspective, the first thing to consider would definitely be no negative side effects of the therapy… After a free try out, I would see if the child is happy after the therapy, and make sure he wasn't irritated by the therapy.” Parent B said, “I would make sure the child is willing to get involved with the therapy, make sure she wasn't resistant to the therapy.”

The teachers in the focus group also reflected on similar traits. Teacher B further suggested that when parents recognize the child's problem, they are more likely to seek help from professionals.

Regarding the factor “access of knowing the therapy,” it has been demonstrated before that the only access reported by the participants of knowing the emotional support service for children was from a friend who works in the field. Parent A further explained how she thought if the access of the therapy was from a friend, the therapy would be trustworthy. She would not try the therapy if a stranger introduced them to her. She said “if a person comes out of nowhere and asks me to try a [form of] therapy I have never heard of, I would not do it. I tried dance therapy because there was a teacher who suggested therapy might be helpful… It is important for me to understand the general rationale behind the therapy through a discussion with the therapist.” This may have further indicated the perception of a lack of knowledge about the access to trustworthy emotional services has limited some Chinese parents' choices of professional emotional support services.

Regarding parents' decisions about play therapy, aside from the common traits discussed before, two new traits were derived: “busy with work” and “lack of knowledge of play therapy.” In the focus group, Teacher C mentioned a perception about play therapy: that it involves more time than the ways children normally play. It was shown under the theme “perception of play,” some Chinese parents find the time commitment of playing with children can be hard to achieve. If the parents also think play therapy cost them more time than play, the trait “busy with work” also indicates the time commitment of participating children's play therapy can be a barrier for parents to be involved with their children's play therapy.

**Figure 11:**
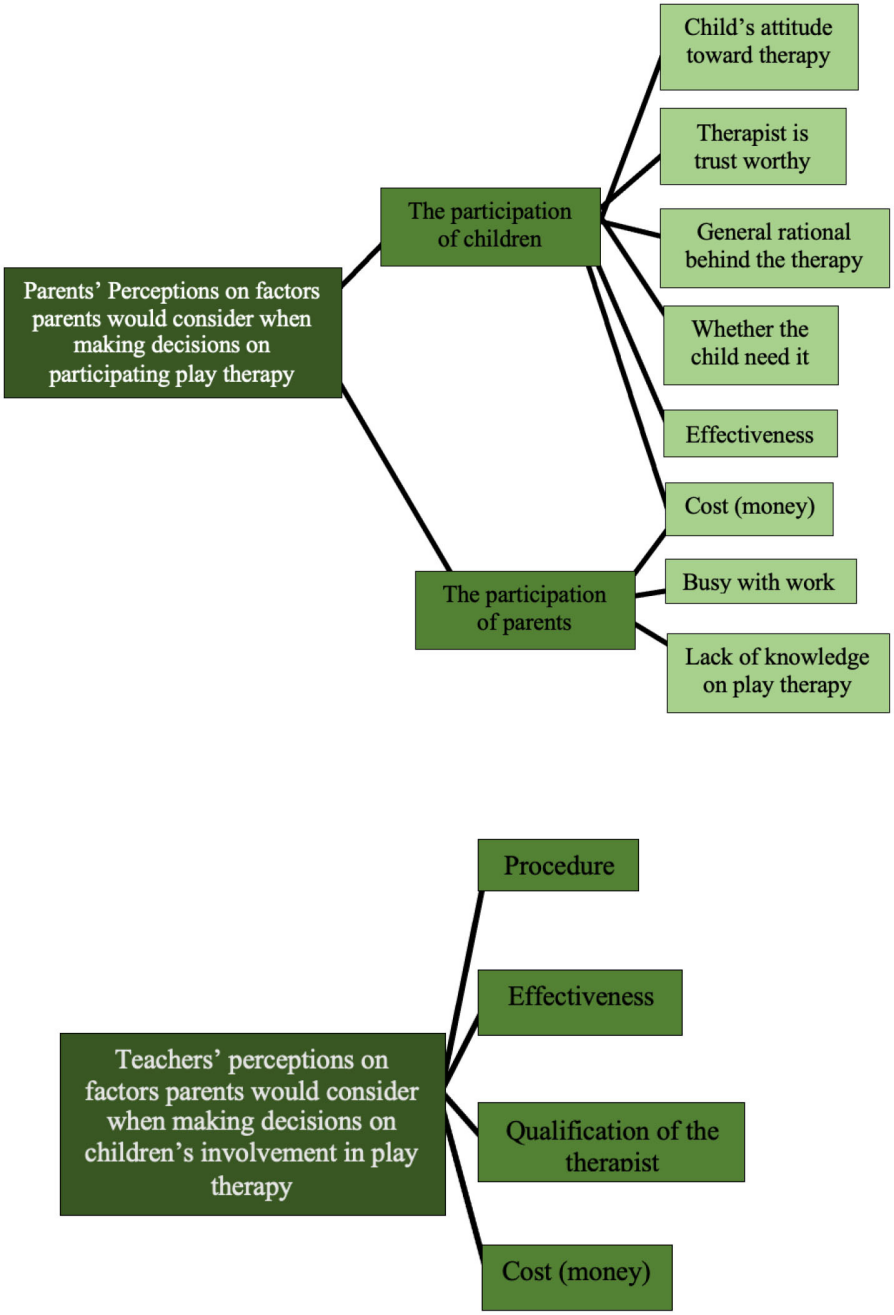
Subthemes and further subthemes for theme 4: factors parents would consider when making decisions on participating in play therapy.

The summary of subthemes and further subthemes derived from the theme of perception on therapy and play therapy that appeared from the responses of the parents and teachers are presented in Figure 11.

## 4. Discussion

### 4.1. Perception of play

#### 4.1.1. Subtheme: time and materials of play

In this study, it was found that both the parents of children who have entered primary school mentioned that schoolwork limits their children's length of time spent on play and the types of materials they play with. This may have indicated that schoolwork caused a reduction of time spent on play, which in turn caused a reduction of materials children play with.

It was also found that Parent A and Parent B (Supplementary Table 1), whose children are younger, tend to limit children's access to electronic devices. This could be related to parents trying to protect their children's eyes. The Ministry of Education of the People's Republic of China (MEPRC) published the ‘Guidance of Learning and Development of Children from Three to Six ‘(GLDC) in 2012. This document set specific goals for limiting the time children should spend watching television etc. to protect their eyes (Ministry of Education of the People's Republic of China., 2012). This shows that protecting young children's eyes is important in the context of China. Furthermore, Lester and Russell (2008) demonstrated that there is evidence showing that electronic devices are associated with less physical activity, which in turn causes some health risks. Another reason for parents limiting children's time spent on electronic devices could be trying to let children have more time for physical activities. However, the results from the interview showed all three children spend most of their time playing at home; thus, within the sample size for this research, protecting the children's eyes seems to be a more suitable reason for limiting children's use of electronic devices.

#### 4.1.2. Subtheme: who children play with and where to play

The results from the interviews indicated that formal education limits children's time spent playing with peers after school. Ginsburg (2007) suggested that formal education in the US comes with busy lifestyles and academic commitments, which limits children's free time to play. The findings in this research may show that formal education had similar influence on children's time of play in China. The limiting effect of schoolwork on play can also be explained by Shen (2016)'s argument. He stated that “once their formal education starts, parents tend to discourage play and focus more on children's academic performance” (Shen, 2016, p. 332). Another possible interpretation of the result could be that some Chinese parents highly value children's academic performance and they would try to limit the children's time and the materials of play for a better academic performance.

It was found in the interviews that economic pressure has influenced the time parents spend playing with children. Previous research has also shown that parents having busy working lives is one of the reasons for limiting the time spent with their children. Gleave (2009) demonstrated the evidence of parents being pressured by their economic status, which limited their time available to play with their children, and which supported the findings of this research. Furthermore, the limited time parents spend with children could also limit children's time spent on outdoor play after school. Parent A suggested her child would have a chance to go out and play with peers when the parent has time to take the child out, but the parent was usually very busy. This is reinforced by Lester and Russell (2008), who have demonstrated the current perception of seeing the outside world as unsafe for children. That means there is a possibility that some parents think that children should only play outdoors under parents' supervision for safety reasons.

It was found in the focus group that children would spend time playing out doors and indoors in kindergarten. According to Ginsburg (2007), outdoor play is related to the benefits of learning life skills, and children's social-emotional development. Giving children the opportunities to spend more time playing outdoors and playing with peers could be beneficial for their social-emotional development, especially when children's time spent on outdoor play and playing with peers are limited.

#### 4.1.3. Subtheme: importance of play

Results from interview and focus group both reported the belief of play being important for children due to the benefits play can bring to children's learning, and psychological health. This view has also been shown in research undertaken by Lin and Yawkey (2013), which showed that Taiwanese parents agreed that play benefits children in their learning skills (problem-solving skills, thinking abilities, etc.) and social-emotional development. The teachers suggested play can benefit them in designing activities and evaluating children's abilities. This view is supported by Hedges et al. (2011), who demonstrated that children's interests revealed from free play are usually used as a source of early years curricula. These benefits of play revealed from the interviews and focus group complement the evidence demonstrated in the section showing the benefits of play for children in the introduction chapter.

In the interviews, Parent A, whose child is in kindergarten, focused on the benefits related to children's learning. Both parents of children in primary school focused on how play can balance the stress that comes with schoolwork. According to Gleave (2009), the increase in academic pressure is associated with the age of children, and the wider policy changes. Zhao et al. (2015) further illustrated the enormous academic stress Chinese students and families experience once the children enter the formal education system due to the highly competitive national college entrance exam in China. Zhao et al. (2015) also demonstrated the negative influence of the enormous academic stress on children's social-emotional status. Looking at academic stress in the context of China, this finding may have shown that the two parents are aware of pressure on children that comes from learning, and they valued not only the children's academic performances, but also their psychological health that is likely to be negatively influenced by formal education. These findings in the current research have also underlined the possibility that some parents' ways of valuing children's learning and emotional health changes after children enter formal education, and that this is due to their awareness of the increased academic stress children are facing in formal education system.

### 4.2. Theme 2: awareness of children's emotional needs

#### 4.2.1. Subtheme: children's styles of expression

This result of this study supported previous research that revealed that happiness was recognized better than the expression of other emotions (Goeleven et al., 2008). An explanation for the result could be that emotions like anger and happiness are more likely to involve social interactions (Elfenbein et al., 2007), so people are more experienced in interpreting these emotions.

It was also shown that parents believed they were able to interpret their children's emotions. They usually interpreted children's emotions through observations of children's facial expressions, behavior and verbal expressions. This might indicate that a large number of children within the sample size expressed their feelings through facial expressions, verbal expression or behaviors. This finding is in alignment with previous research, which demonstrated children would develop various patterns of expression through facial and verbal expressions or behaviors (Chaplin and Aldao, 2013). This could also suggest that the majority of parents and teachers within the sample possessed the ability to interpret their children's emotions by observing their behaviors and verbal expressions.

The results from questionnaire indicated that for some parents, most of the emotions of children in primary school can be relatively easy to interpret, in comparison to the emotions of children in early years education. This result might be explained by the social-developmental theories (Brody and Hall, 2017), which demonstrated how children's emotional expression skills develop over time. Older children's emotions may be easier for people to interpret because they have developed better emotional expression skills than younger children. Another interpretation could be that with parents spending more years with children in primary school age, the parents are gaining more experiences in interpreting the child's emotions. Due to the lack of research into the parents' awareness of their children's emotions, more evidence related to this topic would need to be further researched.

It was shown from the questionnaire that from the perspective of the parents within the sample size, children in early years express their feelings more frequently than children in primary school. Within the sample size, children in early years are more likely to express their feelings through behavior and verbal expression than children in primary school. This result could be caused by the gender differences in children in each age group. Within the sample, four of the five early years children are girls; seven of the ten primary school-aged children are boys, and the only middle school-aged child is a girl. With the majority of the children in each age group being either boys or girls, gender could be a strong influence on the results. Since there is only one parent of a child of secondary school age, any results related to the secondary age group children should be re-evaluated in future research, with a larger sample size.

It was shown in the questionnaire that most girls' emotions (sadness, anger, happiness) are easier to interpret than the boys' emotions. One of the reasons for this result could be related to the frequency of boys and girls showing emotions. According to Chaplin and Aldao's research (Chaplin and Aldao, 2013), girls tend to show more of their happiness and sadness, while boys tend to show more of their anger. The result of this research seems to conflict with the research undertaken by Chaplin and Aldao (2013) showing that girls' anger is easier for the parents to interpret. This could also be caused by the influence of parents' different awareness of boys' and girls' emotions. Endendijk et al. (2017) suggested gender roles caused stereotypical ideas which in turn resulted in different treatment toward boys and girls. Therefore, gender stereotypes could cause different parenting styles in parenting boys and parenting girls. Endendijk et al. (2017) further suggested that parents of girls would be more likely to focus on affiliation and interpersonal closeness.

In Figure 5, it may have shown that within the sample size, from the parents' perspective, girls are more likely to express their feelings through verbal expression or behaviors. Boys are more likely to not express their feelings. This result is supported by Chaplin and Aldao (2013), who suggest that due to gender stereotypes, children are more likely to learn from the caregivers, and to follow the social expectations ascribed to boys and girls. Based on the common gender stereotypes across culture, girls are expected to show emotions more frequently than boys (Brody and Hall, 2017).

#### 4.2.2. Subtheme: possible cause of hard feelings for children

The results from the interviews showed the parents' awareness of how their emotions can influence children. It may also show how some Chinese parents think they could be responsible for their children's feelings. Shen et al. (2018) supported this perception by suggesting Chinese parents feel guilty about their parenting if their children have psychological problems. Chinese parents are likely to see their children's emotional states as part of their responsibility in child-rearing.

#### 4.2.3. Subtheme: important emotional support

Regarding the topic of emotional support for children, the results from the questionnaire indicated that going to a therapist is stigmatized. It may also indicate people may not understand or be familiar with therapy. This may be reinforced in the findings that show parents think therapy is only needed when the child has problems unrelated to day-to-day health. Relevant findings are further discussed in the next section.

In the interviews, it was showed that fewer parents see teachers as important emotional support for children might be related to how teachers are seen as more formal figures who support children with learning, instead of supporting children in social and emotional aspects. This social expectation of teachers is supported by Liu and Meng (2009). In their research, from the perspective of students and parents, positive qualifications are all related to teaching skills and quality of students' learning. None of the characteristics related to emotional support were found in the research. The fact that fewer parents think peers are important emotional support might be related to the data collected about play. It was mentioned above that the three children rarely play with peers after school. Parents are usually with children after school, so they may be less likely to see how peers may have supported their children. Furthermore, there is evidence showing that parents are more likely to provide children with emotional support than peers and teachers. Chaplin and Aldao (2013) demonstrated that children would feel more comfortable showing any emotions in front of their parents than other people, so parents had a greater chance of seeing their child expressing negative emotions. This means parents are more likely to know when children are facing difficult feelings, and when children may need emotional support.

In the focus, group, the three teachers ranked parents as the most important emotional support for children, and teachers are ranked second, peers are ranked the third. This ranking was different from the results of the questionnaire, in which more parents see peers as important emotional support than teachers. This difference in the rankings of emotional support from parents and emotional support from teachers may be caused by the different backgrounds of the participants. All three teachers work in kindergarten, and most of the parents who participated in the research are parents of children in primary school. People may have different expectations for kindergarten teachers and teachers working in the formal education system. According to Cui et al. (2016), Chinese parents' expectations for a good kindergarten teacher are likely to be related to “love” and “respect” to children. These are also competencies of teachers listed in “Ministry of Education (2012).”

Teachers in the focus group also indicated the possibility that with teachers' direction, peers can be important emotional support for children in kindergarten. The teachers' perspective on children's emotional support are supported by Pech (2013) who found that the development of positive peer relationships was built upon the positive relationship between kindergarten teachers and students.

### 4.3. Theme 3: perceptions of therapy and play therapy

#### 4.3.1. Subtheme: perceptions of therapy

It was shown from questionnaire, interviews and focus group that most the participants are lack of knowledge in therapy, possess negative perceptions in mental health services and often think therapy is only for people with disorders. Shi et al. (2020) suggested that most Chinese people are reluctant to make use of mental health services. According to Wang et al. (2019), western parents with negative perceptions of mental health services are less likely to seek help from the services. The negative perceptions toward therapy found in this research may indicate another reason why Chinese people are reluctant to engage with therapy.

Regarding the circumstances children may need to be engaged with therapy, it was indicated that some parents think therapy is for children who act very differently to other children, while fear of the dark may seem to be normal for most of children from the perspective of parents, so children are in this case not considered to be in need of therapy to treat it. This result was supported by Merikangas et al. (2011), who found out American families with children experiencing more severe mental health problems are more likely to get treatment from professional therapists. This may also have indicated that the severeness of children's mental health problems could also be an indicator for Chinese family accessing the therapy from professionals.

#### 4.3.2. Subtheme: perception of play therapy

In alignment with the previous findings on important emotional support for children, the parents think play therapy for day-to-day health should be conducted by parents instead of professional therapists (Supplementary Table 13). This may indicate that the two parents see play therapy conducted by parents as casual activities between parents and children and they may think of it as a way to prevent future problems by constructing healthy parental relationships. This may indicate that some Chinese parents may be willing to be involved with filial therapy, even if they identify their children as having no mental health issues.

Furthermore, this perception of play therapy sounds similar to the filial play therapy approach. Filial therapy is a process of training and supervising parents or other caregivers in child-centered play therapy skills (Lindo et al., 2016). According to VanFleet (2014), in this process, therapists try to help parents develop skills in understanding their children's play themes and their emotions. The parents are then able to understand their child through interpreting their play themes and emotions and they may be able to provide the support and make changes based on the child's needs. Moreover, according to Bifulco and Thomas (2012), through the process of filial therapy, children would not only have a chance to develop a healthier attachment to their parents, but also the parents may overcome any attachment issues they may have. That means filial therapy is not only beneficial for children, but also beneficial for the parents. Only the benefits of play therapy for children were revealed in this research, and none of the participants talked about the benefits for parents when discussing the advantages of play therapy in the interviews. One possible explanation for this is that the participants perceived the focus of the research to be on the benefits of play therapy for children, and they were not specifically prompted to discuss any benefits for parents during the interviews. Another explanation for this result could be that the parents did not perceive any direct benefits of play therapy for themselves. Future research could explore parents' perceptions of play therapy with children and whether it could contribute to the possibilities of parents and their children being involved in play therapy.

It was reflected in the interviews that some parents may think they have the ability to conduct play therapy to their children without any training as long as they were able to recognize their children's emotional needs. Different from the parent's perception, Bifulco and Thomas (2012) demonstrated that the individual who conducts the play therapy for children should be carefully considered, because the person who conducts play therapy should be decided based on the type of problems the children are facing, which should be identified by professionals. That means, in the process of filial therapy, professionals are still necessary in training caregivers and identifying children's problems. This may indicate that for some Chinese parents, the more confident the parents are in their abilities to recognize their children's emotional needs, the less likely they would be to consider becoming involved with filial therapy.

#### 4.3.3. Subtheme: perception on the cost of therapy/play therapy

This study indicated that the cost of the therapy could be another factor limiting Chinese parents' access to seek help from mental health services. This finding is in alignment with the findings of American parents' perceptions on mental health services found by Reardon et al. (2017) and Hronis et al. (2020) (See Introduction).

The effectiveness of play therapy was recognized by the parents in the interviews. However, as mentioned before, their negative perceptions on therapy may reduce the possibilities of some Chinese parents letting their children engage with play therapy with professionals. Further details on other factors that may influence parents' decisions on the engagement of play therapy are discussed in the next section.

### 4.4. Theme 4: factors influencing parents' decisions about engaging with play therapy

The traits revealed from focus group and interviews under this theme may indicate that Chinese parents' ability to recognize their children's problems can be an indicator of the parents letting their children engage with play therapy. This finding is supported by Sayal et al. (2006), who found the same correlations in Western families. Teacher C suggested that in rural areas, parents rarely seek help from professionals, even when the child may have mental health issues. She further suggested the cost of the services could be the reason. This result may indicate that the living area may be a factor related to some Chinese parents' economic status, which in turn reduced the possibility of the parents seeking help from professionals. Similar to this finding, Morales et al. (2020) found that Western families living in urban areas are more likely to seek help from emotional support services for children.

It was also revealed that the parents are lack of trust worthy access to therapy could be another barrier of parents seeking help from mental health professionals. This finding is aligned with the results from research undertaken by Reardon et al. (2017), in which they found out Western parents' limited knowledge in the process of seeking help for their children's mental health problems could be a barrier. In terms of the factor “whether the child needs it,” this finding aligns with research carried out by Hronis et al. (2020), which indicated how therapy can be useful for children's needs as one of the factors parents would consider when seeking help from mental health services for their children. This trait may also be related to the common trait of perception of therapy and play therapy as being “only for people with disorders.” When considering letting children try play therapy with a professional therapist, some parents may think only children with disorders would need it. The idea that therapy is only for people with disorders, and the likelihood of refusing to recognize the child might have social-emotional problems, this perception may also limit some Chinese children's access to play therapy. As mentioned above, even if some Chinese parents do agree that play therapy may be needed to prevent future problems, the cost of seeking professional help for day-to-day health could be a barrier.

The barrier caused by the perceived time commitment, and lack of knowledge in mental health services for children was found in this study. This finding is in align with previous research on Western parents' perceptions (Reardon et al., 2017). Comparing the findings of barriers of Chinese parents letting their children engage with play therapy in this research and barriers of Western parents seeking help from mental health services for children, the findings supported each other; this may show there are no cultural differences between Chinese and Western parents' perceptions of the barriers.

## 5. Conclusion

### 5.1. Back to the research questions

#### 5.1.1. What are Chinese parents' and teachers' perceptions on the role of play and how may this connect to parents' decisions about letting their children engage with play therapy?

It was found that the parents and teachers in this research all had a positive perception of play, which is related to the benefits of play in children's learning and psychological health. These positive perceptions of play may be related to their positive perceptions on play therapy, in which they think play therapy can be effective in maintaining children's mental health and solving pre-existing mental health problems. These positive perceptions may encourage some parents' willingness to try play therapy. The influence of school was found to limit children's time spent on play. The parents' time spent with children was also found to be limited by parents being busy with work. This may also indicate that the commitment of time on play therapy can be a barrier for parents and their children to engage with play therapy.

#### 5.1.2. What are the Chinese parents' and teachers' perceptions on children's emotional needs and how it may be related to parents' decisions about letting their children engage with play therapy?

The parents and teachers who participated in this research are confident about their abilities of recognizing children's emotions and children's mental health states. The parents and teachers all have an awareness of taking responsibility in supporting children emotionally. This confidence in the ability of recognizing and supporting children emotionally may reduce the possibilities for the parents to seek help from professionals and letting children to be involved with play therapy because the parents may think therapists are not needed when they themselves are sufficient in emotionally supporting their children.

#### 5.1.3. What are Chinese parents' and teachers' perceptions of therapy and play therapy and how may they be related to parents' decisions about letting their children engage with play therapy?

The perception of therapy and play therapy from Chinese parents' and teachers' perspectives on therapy and play therapy in this research are found to include: lack of knowledge about therapy and play therapy, lack of knowledge about the process of getting help from emotional support services, and mostly negative perceptions on emotional support services in China, such as the high costs and time required, doubts regarding the qualifications of the therapists, etc. These perceptions could be barriers for Chinese parents letting their children engage with play therapy. Furthermore, the perception that only children with severe mental health issues should engage with play therapy conducted by a therapist may also reduce the possibilities of parents getting emotional support for their children. As mentioned before, in the context of China, parents may feel ashamed to admit their child has mental health problems, which may in turn discourage them from seeking emotional support services for their children, including play therapy.

### 5.2. Reflection/limitations of the study and implications for future research

While conducting this research, the policies related to COVID-19 in China limited the number of the participants I was able to recruit for the research. Snowball sampling enabled me to recruit the participants in a short timeframe and conduct the research. However, due to the small and uneven sample size, the research results are not intended to generate theories on the relevant issue. The purpose of the research is to try to understand more possible factors that influence parents' decision in letting their children engage with play therapy. Further research with larger sample sizes will be needed to evaluate the interpretations and the results derived from this research.

When undertaking the focus group interview, the participants seemed to be lacking in focus when getting to the end of the discussion. This may have been caused by the questions becoming more difficult when they are about play therapy, an issue that the participants are not familiar with. For future research, the order of the questions may need to be changed to embed the complex questions in the middle of the questions on the issues the participants are more familiar with, to help them remain interested and focused on the discussion.

## Data availability statement

The raw data supporting the conclusions of this article will be made available by the authors, without undue reservation.

## Ethics statement

The studies involving humans were approved by Institute of Education, University College London. The studies were conducted in accordance with the local legislation and institutional requirements. The participants provided their written informed consent to participate in this study.

## Author contributions

HC: conceptualization and validation, methodology, writing, review and editing, formal analysis, data curation, writing—original draft preparation, investigation, and project administration.
