# Supplementary material for: Analyzing Chinese parents' and teachers' perception of play, children's emotional needs and therapy: implications for seeking help
Source: Front Psychol. 2023 Sep 8;14:1219901. doi: 10.3389/fpsyg.2023.1219901 (PMC10516295; doi:10.3389/fpsyg.2023.1219901)
Supplement: Supplementary file 1 [file Table_1.DOCX]

Supplementary Material

**Perspectives of Parents of Children in China on Play Therapy**

Huiting Cao*

*** Correspondence:** Huiting Cao: qtnzhca@ucl.ac.uk

# Supplementary Tables

Supplementary Table 1. Specific subthemes derived from *Materials for play* and examples from parents’ data set for theme 1: Perception of play.

| Specific Subtheme | Example |
| --- | --- |
| Avoiding electronic devices | Parent B: ‘Normally, she’s not allowed to play electronic devices. She would just play with her dolls, or cards on the table.’ |
| Video games and mobile games | Parent C: ‘He would spend most of the time playing mobile games, and live football (a kind of video game).’ |
| Choice of materials limited by schoolwork | Parent C: ‘Before he also played music instruments and basketball, with the growing of his grade in primary school, he stopped playing the instrument and basketball, and spent more time on studying.” |

Supplementary Table 2. Specific subthemes derived from *People children play with* and examples from parents’ data set for theme 1: Perception of play.

| Subthemes | | Example |
| --- | --- | --- |
| Parents | Busy with work | Parent A: ‘I rarely play with him. I get off work late every day, most of the time, he would fall asleep by the time I arrived home. Recently I tried to get home a couple of minutes before he goes to bed, so we can see each other. Normally, I don’t have time to play with my child.’ |
|  |  | Parent C: ‘not a lot of people would do what we did for the child. I was in HR, and I quit my job, and opened a store. The father also quit his job and wanted to focus on accompanying the child. To us, (parenting) the child is very important, and we want to be there for him when he’s growing up. Most of the parents are not able to do these things.’ |
| Alone | Child’s preference | Parent B: ‘Now, she’s in the first grade of primary school, we don’t spend as much time playing with her as before. She seems doesn’t need the family to always play with her. When she was younger, we played with her more. The adults had to play with her all the time when she was younger…’ |
| Other families | Grandparents and aunt | Parent B: ‘Now, only when playing games like hide and seek, she may play with the grandparents and her aunt.’ |
| Peers | Outdoor | Parent A: ‘…he comes back late from school. When I had time, I would bring him outside to let him play with other kids, but most of the time he would playing Lego at home’ |
|  | At school | Parent B: ‘At school, she would definitely play with other children. But at home, she basically doesn’t play with other kids. The neighborhood, the children are not [living] as close as before. She just plays at home, most of time playing alone.’ |
|  | On weekends | Parent C: ‘On weekends, he would ask other kids to play together, but during weekdays, he goes to school during the daytime, and plays with his dad at home after school.’ |

Supplementary Table 3. Specific subthemes derived from *Where to play* and examples from teachers’ data set for theme 1: Perception on play.

| Subthemes | Example |
| --- | --- |
| Indoor play zones | Teacher C: ‘Like the other teacher just said, during the free play in the classroom, children would go to different play zones. The first thing they do when they come to school is to decide which zone they want to play at.’ |
| Outdoor | Teacher C: ‘During the outdoor play, children would choose which group they want to play with, children will organize their games, for example, these children want to throw sandbags, those children want to play with the castle etc. They have strong autonomy.’ |

Supplementary Table 4. Specific subthemes derived from *Importance of play* and examples from parents’ data set for theme 1: Perception of play.

| Subthemes | | Example |
| --- | --- | --- |
| Benefits of play | Learning | Parent A: ‘it depends on the type of play. If the play is interactive, children can learn how to get along with others through communicating with people. It the play is related to skills, such as sports, children may get physically stronger, better at controlling his body, he may be able to feel more secure; if the play is related to intelligence, by getting in touch with more new materials, he may be able to absorb more information.’ |
|  | Psychological health | Parent B: ‘…[balancing] the pressure comes from the schoolwork, or for psychological health, I think it can be helpful to all of that. I think the child’s ways of thinking can be magnified while playing, this process is helpful to children’s intelligence and mental health.’ |

Supplementary Table 5. Specific subthemes derived from *Importance of play* and examples from teachers’ data set for theme 1: Perception on play.

| Subthemes | Example |
| --- | --- |
| Positive emotion | Teacher C: ‘Play is their favorite thing, if you let them play all the time, they would be so happy!’ |
| Broadening mind/learning | Teacher B: ‘They can understand it better only if you let them actually do it. For example, we plant mushrooms together, and the children would use the mushrooms to pretend to cook them. They would have conversations about how to cook it, what do they need to cook it. I think [play] is beneficial for broadening their mind, and having practical experiences related to real life.’ |
| Reveal children’s abilities and interests | Teacher A: ‘During the free play time, their abilities can be shown in a more truthful way. During the group play time, children may act the way what they think the teachers would want. But during free play, their real abilities can be revealed, so when evaluating children’s development, we usually observe them during the free play time. I think play is also important for activity development. Through the process of play, teachers can trace a child and find out about their interests, so the teachers can design the activities based on their interests.’ |

Supplementary Table 6. Specific subthemes derived from *ways to interpret children’s emotions* and examples from parents’ data set for theme 2: Awareness of children’s emotional needs.

| Subthemes | | Example |
| --- | --- | --- |
| Behavior | | Parent A: ‘When strangers appeared in a familiar place, his body looked stiff, and he seems to not able to do things he could do well before.’ |
| Facial expression | | Parent C ‘He basically doesn’t cry, he seems to be happy every day, he always laughs or smiles, when he’s excited, he would raise his eyebrows toward you.’ |
| Verbal expression | Language tone | Parent B: ‘…she would be like ‘fine! I got it!’ the language tone was very impatient.’ |
|  | Words | Parent C: ‘He would tell you about his feelings in his own language. He would create some new words and say it to you.’ |

Supplementary Table 7. Specific subthemes derived from *Ways of interpreting* and examples from teachers’ data set for theme 2: Awareness of children’s emotional needs.

| Subthemes | Example |
| --- | --- |
| Time of the child staying focused | Teacher B: ‘When doing the evaluations, we would observe, observe the length of time the child is able to focus on an activity. If the child was able to stay focused for a long time, maybe over twenty minutes for a thirty-minute activity, then the child is interested in the activity.’ |
| Facial expression | Teacher A: ‘Most children’s emotion would be expressed through their facial expression and their language.’ |
| Language/ Verbal expression |  |
| Behaviors | Teacher C: ‘When children are not interested in toys or other materials in the classroom, they would stop playing, and sit there, doing nothing.’ |

Supplementary Table 8. Specific subthemes derived from *possible cause of hard feelings for children* and examples from parents’ data set for theme 2: Awareness of children’s emotional needs.

| Subthemes | | Example |
| --- | --- | --- |
| Social situations | Daily observation | Parent A: ‘The daily fear and nervous in some social situations are always hard for him maybe, I think… I’m just saying based on my observation.’ |
| Parents being angry | Personal experience | Parent B: ‘Sometimes, parents would fight, maybe this can be hard for the child to face. Nonetheless, I can completely understand from her point of view, but when I was young, I was afraid of my parents fighting, even after all these years, but thinking from her perspective, I think the influence on her can be pretty big.’ |
|  | Daily observation | Parent C: ‘Maybe [the emotion] comes from how I, as a mother can be emotionally unstable sometimes. I can be a little irritable sometimes, and when I sometimes give vent to my anger (in front of him), it can be hard for him to face… He would cry when it happens, he rarely cries.’ |

Supplementary Table 9. Specific subthemes derived from *important emotional support for children* and examples from parents’ data set for theme 2: Awareness of children’s emotional needs.

| Subthemes | | Example |
| --- | --- | --- |
| Parents | Learning | Parent B: ‘I think whatever we do together, I think the company of the parents is the most important thing for her. She can get the warmth, and learn some life skills, bravery, these things are the things she needs to get from adults…At least when being with her, the adults would show her the positive characteristics, and these positive things can be passed to her.’ |
|  | Communication | Parent C: ‘He would communicate with his father about everything. The main means of communication for them is to play a live football game (a video game). Through playing against each other, they build a secure relationship in which they can communicate with each other. He can’t communicate with his peers about his feelings, because the peers can be very misleading, so he doesn’t tell them what his real thoughts are. For example, his classmates may say ‘this teacher is so annoying, giving us so many homework, I really want to … this teacher, you think so too, right?’ |
|  | Positive feedback | Parent A: ‘Depends on the situation. When I’m around him, he may need me to give him positive feedback, to give him confidence. In other situations, he mainly needs other adults to give him confidence and feeling of secure. I think adults are more helpful to him, maybe because he interacts with teachers more (than with other kids) … He doesn’t have many chances to interact with peers, he is also not interested in doing it. So I think, to him, peers are not that important, he may not be able to get emotional support from peers. Of course, this is my guess based on my observation.’ |
| Teachers | Positive feedback |  |

Supplementary Table 10. Specific subthemes derived from *Important emotional support* and examples from teachers’ data set for theme 2: Awareness of children’s emotional needs.

| Subthemes | Example | |
| --- | --- | --- |
| Parents | Teacher A: ‘After a child is born, an attachment will be built up between the child and the parents. If the child had a secure attachment with the parents, then when the child enters kindergarten, she/he would be able to adapt to the new environment and the new people soon. And then a relationship will be built between the child and the teacher in the kindergarten, the teacher would help the child to transit the anxiety of leaving the parents… And then the child would be able to build up friendships with peers.’ | |
| Teachers |  |  |
| Peers |  |  |
| Psychological therapist | For targeted people | Teacher B: ‘I think a therapist is for children with extreme behaviors. Normally, if we recognized some minor social-emotional problems, we would communicate with the parents. If the parents would work with us on it, the problem would be reduced… But when severe problems occurred, if the parents realize the problems after communicating with the teacher, they would hire professional therapist for the children…Some parents just don’t realize social-emotional difficulties can be a problem for children…’ |
|  | Limited by economic status | Teacher C: ‘Our kindergarten is a county kindergarten, the parents we work with are mostly from rural areas. Sometimes we realize a child is behaving in an abnormal way and we recommend to the parents to get help from a professional therapist. But maybe because the parents can’t afford the cost of it, they would just beg us to help the children, to pay more attention to the child.’ |

Supplementary Table 11. Specific subthemes derived from *Perception on therapy* and examples from parents’ data set for theme 3: Perceptions of therapy and play therapy.

| Subthemes | | Example |
| --- | --- | --- |
| Lack of publicity | Having psychological problems | Parent A: ‘I think it’s only related to the targeted population. If the child had certain problems related to psychology, no matter if it is mild or severe, the parents of the child may have heard of the word “therapy”. If the person hasn’t got a chance to have a deep discussion with the professionals about what the therapy is, it might be hard to understand what it is.’ |
|  | Access to Psychology courses at school | Parent B: ‘... Most people rarely engage with therapy conducted by professional therapists, so people don’t know about the specific methods about the procedures of psychological therapy. Unless they had psychology courses at school…’ |
|  | Access to relevant trainings at work | Parent C: ‘In China, therapy is not a very well-known word. Most people work in fields that are not related to it at all. If one’s job is related to therapy in anyway, the person would at least have some understanding in therapy.’ |

Supplementary Table 12. Specific subthemes derived from *Perception on therapy* and examples from parents’ data set for theme 3: Perceptions on therapy and play therapy.

| Subthemes | Example |
| --- | --- |
| Not sure on how effective the therapy is | Parent A: ‘Generally, I don’t have negative feelings toward the experience. It’s just that I’m not sure how effective the therapy is. My child is having different training at the same time, some are physical training, some are psychological trainings, like the dance therapy I have talked about. I can tell my child is getting better on his social emotional problems, but I don’t know which training is more effective, and which one is not very effective, I just know the combination of the training seems to work on my child. ’ |
| Doubts on the qualifications of the therapist | Parent C: ‘The training I had was very bad… The therapist seemed to be very unprofessional, and it was not worth the money…’ |
| Only for people with disorders | Parent B: ‘I don’t have any relevant experiences related to therapy. I’ve always thought therapy is just for people with psychological problems. Those people would need therapy to solve certain targeted problems.’ |

Supplementary Table 13. Specific subthemes derived from *Who should conduct play therapy* and examples from parents’ data set for theme 3: Perceptions of therapy and play therapy.

| Subthemes | | Example |
| --- | --- | --- |
| Parents | Daily activities | Parent B: ‘Parents who have enough time to learn about play therapy and have time and patience to spend time with children have the ability to conduct play therapy… I think parents would be better to conduct play therapy as children’s daily activities. If teachers are trying to carry out the therapy, they can only do the therapy in one setting, in the school. It’s impossible for us to go to a professional organization for play therapy, because our child is healthy. So only parents can use the therapy in various settings, this would make the therapy more efficient.’ |
|  | Patient to get along with children |  |
|  | Have time to learn about play therapy |  |
|  | Know the child well | Parent C: ‘My child’s father is doing play therapy with my child. He was able to do it because he knows the child well. He knows what the child is thinking no matter if he is talking, or not talking (about his thoughts). And the father was able to interpret the child’s facial expressions.’ |
| Professional therapists | Treat psychological problems | Parent C: ‘if the child has social-emotion problems, the more professionalized play therapy may be needed, and a professional therapist should be the one to conduct the therapy to help the child with social emotional problems.’ |

Supplementary Table 14. Specific subthemes derived from *What is play therapy* and examples from parents’ data set for theme 3: Perceptions on therapy and play therapy.

| Subthemes | Example |
| --- | --- |
| Treat psychological problems | Parent B: ‘when the child is facing social emotional problems, play therapy is a way to help the child to solve the problems.’ |
| Daily activities for children | Parent C: ‘I don’t think children have to have had experience of trauma in order to be engaged play therapy. If the tools or the games you use or in the therapy attracts children, then the children who get to do play therapy shouldn’t be limited… It can be a kind of daily activity for children to maintain their psychological health.’ |
| Build up confidence | Parent A: ‘I feel the game therapy may be similar to dance therapy my child is doing right now. There would be a main activity, it’s also a kind of game to me. Using different kinds of teaching tools, building a relationship with the teacher, the teacher can gain children’s trust. Activities can be related to physical balance, or accomplish some mission, and these activities can help children build up confidence.’ |
